# Baricitinib reverses HIV-associated neurocognitive disorders in a SCID mouse model and reservoir seeding in vitro

**DOI:** 10.1186/s12974-019-1565-6

**Published:** 2019-09-27

**Authors:** Christina Gavegnano, Woldeab B. Haile, Selwyn Hurwitz, Sijia Tao, Yong Jiang, Raymond F. Schinazi, William R. Tyor

**Affiliations:** 1 Laboratory of Biochemical Pharmacology, Department of Pediatrics, Emory University, Atlanta, GA 30322 USA; 2 Emory Center for AIDS Research (CFAR), Emory University, Atlanta, GA 30322 USA; 3 Department of Neurology, School of Medicine, Emory University, Atlanta, GA 30209 USA; 4 Atlanta Veterans Affairs Medical Center, Decatur, GA 30033 USA

**Keywords:** HIV-associated neurocognitive disorders, JAK inhibitor, Baricitinib, Neuroinflammation, Object recognition testing, Mononuclear phagocytes

## Abstract

**Background:**

Since HIV-associated neurocognitive disorders (HANDs) occur in up to half of HIV-positive individuals, even with combined antiretroviral therapy (cART), adjunctive therapies are needed. Chronic CNS inflammation contributes to HAND and HIV encephalitis (HIVE). Baricitinib is a JAK 1/2 inhibitor approved in the USA, EU, and Japan for rheumatoid arthritis, demonstrating potent inhibition of IL-6, D-dimer, CRP, TNF-α, IFN-α/β, and other pro-inflammatory cytokines.

**Methods:**

Our modified murine HAND model was used to evaluate the ability of baricitinib to cross the blood-brain barrier (BBB) and modulate monocyte/macrophage-driven HAND. Severity of HAND was measured by assessing cognitive performance of low- and high-dose baricitinib treated versus untreated HAND mice. The severity of brain neuroinflammation was evaluated in these mouse groups after flow cytometric analyses. We also assessed the ability of baricitinib to block events in myeloid and lymphoid cells in vitro that may undergird the persistence of HIV in the central nervous system (CNS) in primary human macrophages (Mϕ) and lymphocytes including HIV replication, HIV-induced activation, reservoir expansion, and reservoir maintenance.

**Results:**

In vivo, both doses of 10 and 50 mg/kg qd baricitinib crossed the BBB and reversed behavioral abnormalities conferred by HIV infection. Moreover, baricitinib significantly reduced HIV-induced neuroinflammation marked by glial activation: activated microglia (MHCII^+^/CD45^+^) and astrogliosis (GFAP). Baricitinib also significantly reduced the percentage of p24+ human macrophages in mouse brains (*p* < 0.05 versus HAND mice; *t* test). In vitro, baricitinib significantly reduced markers of persistence, reservoir size, and reseeding in Mϕ.

**Conclusion:**

These results show that blocking the JAK/STAT pathway reverses cognitive deficits and curtails inflammatory markers in HAND in mice. Our group recently reported safety and tolerability of ruxolitinib in HIV-infected individuals (Marconi et al., Safety, tolerability and immunologic activity of ruxolitinib added to suppressive ART, 2019), underscoring potential safety and utility of JAK inhibitors for additional human trials. The data reported herein coupled with our recent human trial with JAK inhibitors provide compelling preclinical data and impetus for considering a trial of baricitinib in HAND individuals treated with cART to reverse cognitive deficits and key events driving viral persistence.

**Electronic supplementary material:**

The online version of this article (10.1186/s12974-019-1565-6) contains supplementary material, which is available to authorized users.

## Introduction

Of the estimated 38 million people living with HIV-1 infection, HIV-associated neurocognitive disorders (HANDs) occur in up to 50%, even with combined antiretroviral therapy (cART) [1–4]. Animal models that recapitulate behavioral and phenotypic events that define HAND have proven essential to investigating the correlate mechanism(s) of disease and development of novel therapeutic strategies [5–9].

Previously, we established a murine HAND model that comprises intracranial (IC) injection of HIV-1 infected human monocyte-derived macrophages (MDM) into a SCID mouse with primary readouts of cognitive dysfunction measured by maze learning and pathological indices quantified with immunohistochemistry that included amounts of astrogliosis and mononuclear phagocytes and decreases in MAP2 immunostaining (a marker for arborization of neuronal dendrites) [6–9]. There was little to no evidence of neuronal death. Importantly, the model now incorporates object recognition testing (ORT) to demonstrate mild behavioral deficits in HAND mice prior to and after treatment with novel agents [10]. Of note, the ability to detect milder forms of HAND is particularly relevant for early intervention strategies, because it is believed that delayed detection of only the more severe HAND (e.g., HIV-associated dementia) is less likely to respond to treatment.

Chronic inflammation contributes to HAND and HIV encephalitis (HIVE) and is hallmarked by elevated levels of IL-6 and other pro-inflammatory cytokines in vivo*,* as well as elevated cellular inflammatory markers including CD163 and CD14/CD16 [8, 11–13]. Since our previous work showed that ruxolitinib, a JAK inhibitor, ameliorates HIVE in our mouse model [8], we hypothesized that baricitinib, a once a day oral FDA-approved JAK 1/2 inhibitor, would impact these inflammatory markers without affecting immune responses necessary to control infection and would reverse cognitive deficits. Baricitinib is approved for chronic long-term use without a black-box safety label. Thus, we used our HAND model to evaluate baricitinib and to demonstrate its beneficial effect on brain inflammation and ORT. We simultaneously evaluated the effect of baricitinib on key events driving viral persistence using primary human lymphocytes and macrophages (Mϕ), including HIV replication, HIV-induced activation, reservoir reseeding, and reservoir maintenance in vitro. Together, we demonstrate that baricitinib crosses the blood-brain barrier (BBB), reverses ORT abnormalities, and decreases HAND/HIVE pathological markers in a SCID mouse HAND model. Baricitinib significantly reduces markers of persistence, reservoir size, and reseeding in Mϕ in vitro. This preclinical data suggests that baricitinib could reverse HAND in patients and key events driving viral persistence, including downregulation of soluble and cellular markers in myeloid cells that promote viral trafficking to the central nervous system (CNS), mononuclear phagocyte inflammatory activity with the CNS, and reservoir seeding peripherally and across the CNS including sCD163, sCD14, IL-6, D-dimer, CD14/16, HLA-DR, PD-1, and Bcl-2. Our group has recently completed A5336, an ACTG-funded multisite phase 2a study in humans to assess the safety and efficacy of ruxolitinib, a similar JAK 1/2 inhibitor; data were reported by Marconi et al. [14], underscoring safety and potential efficacy; therefore, consideration for baricitinib for milder HAND is warranted and contextually relevant. Our studies provide a strong impetus for clinical trials of baricitinib in persons with HAND receiving cART that build upon existing human trials and the data reported herein.

## Methods

### Human monocyte-derived macrophages and infection with HIV-1 for murine studies

Human MDMs were generated and routinely cultured as described previously [7, 8, 15]. A Mϕ-tropic HIV-1 viral strain (HIV-1_ADA_) was selected for routine infection of the MDMs in vitro; this strain was used because it has been validated in our in vivo model and constitutes an M-R5 Mϕ-tropic strain suitable for infection in Mϕ. Both the primary human monocytes and HIV-1_ADA_ were obtained from Dr. Howard Gendelman (University of Nebraska Medical Center, Omaha, NE). Briefly, monocytes were stimulated with 7 ng/mL Mϕ colony-stimulating factor for 7 days to generate MDMs, followed by HIV-1 infection (MOI of 0.1) for two more weeks to allow for sufficient infection of at least approximately half of the cells. MDMs were collected and re-suspended in sterile phosphate-buffered saline (PBS) for intracranial (IC) inoculation.

### Animals

Five-week-old B6.CB17–Prkdc/Szj (SCID) male mice are purchased from Jackson Laboratory (Bar Harbor, Maine, USA), singly housed in sterile microisolator cages, and given 1 week to acclimate before experimentation. All protocols were approved by the Atlanta VA Institutional Animal Care and Use Committee.

### Animals and inoculation

**Fig. 1:**
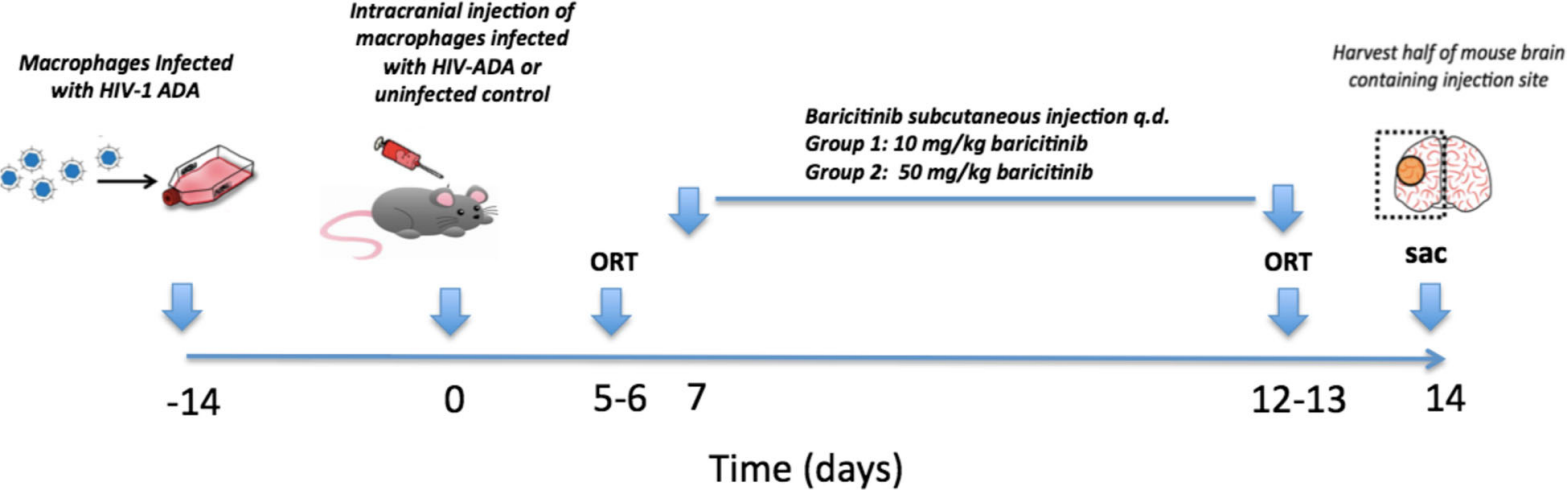
Schematic diagram showing the SCID mouse HAND model and ORT paradigm used

Four-week-old B6.CB17-*Prkdc*^scid^/SzJ (SCID) male mice were injected IC with 30 μL of HIV-infected MDMs suspended in 0.9% saline, directly into the frontal lobe of the right hemisphere. The injection was done free-handed (without a stereotactic device). The injection is performed with a syringe that allows a very slow rate of infusion. We have verified the injection site with a histological staining in our previously published works, which is in the basal ganglia as described in Koneru et al. [16]. Injections were performed on day 0 (D0). Treatments (baricitinib or saline) were given subcutaneously (SC). There were 4 groups of mice: (1) control (uninfected MDM) group, (2) HIV-infected MDM (HAND) plus saline, (3) HIV-infected MDM (HAND) + 10 mg/kg baricitinib qd, and (4) HIV-infected MDM (HAND) + 50 mg/kg baricitinib qd (*n* = 9 per group). Overall study design is summarized in Fig. 1.

### Quantification of baricitinib in the murine brain and drug dosing calculations

To measure the pharmacokinetics of baricitinib in the mouse brain and plasma, the drug was administered SC to SCID mice (*n* = 3 for each time point). To select murine dosages for this study, we performed a mathematical calculation for species scaling using traditional two-compartment pharmacokinetic dynamics, volume of distribution, and rate of elimination to model a murine dose of baricitinib that is equivalent to the human 2 mg or 4 mg qd dosing, which are approved doses in humans (FDA approved, and European and Japan approved, respectively). There are currently 21 studies underway in the USA evaluating the safety and efficacy of baricitinib for 4 mg qd dosing, warranting consideration for species-scaled 4 mg qd dosing in our study (ClinicalTrials.gov). Ten milligrams per kilogram (species scaled to human 2 mg qd dose) and 50 mg/kg (species scaled to human 4 mg qd dose) of baricitinib were dissolved in delivery medium (2% DMSO + 30% PEG + 5% Tween 80 + ddH2O). One, 4, 8, and 24 h after drug inoculation, mice were sacrificed and whole blood was collected immediately followed by cardiac perfusion with PBS prior to the extraction of brain tissues. After brain extraction, posterior fossa (the cerebellum and brainstem) was taken, snap frozen, and stored at − 70 °C. Posterior fossa from each mouse was then weighed and homogenized in a mixed water and methanol (2:1) solution. The homogenates were then centrifuged, and the pellet discarded. The supernatant was dried by evaporation using nitrogen gas, resuspended in 1 mL water, and put through Isolute-XL C18 columns (Biotage, Uppsala, Sweden). Drug was then eluted with acetonitrile and subsequently dried. Finally, samples were resuspended in 1 mL of 75% methanol (containing 0.1% formic acid), and baricitinib levels were measured by liquid chromatography and tandem mass spectrometry (LC-MS/MS). Chromatographic separation was performed by Ultimate 3000 LC system (Thermo Scientific, MA, USA), on a Kinetex XB-C18 column (50 × 2.1 mm) with a 2.6-μm particle size (Phenomenex, CA, USA). The mobile phase A consisted of 0.1% formic acid in water, and the mobile phase B consisted of methanol. Isocratic elution was used for the separation with mobile phase A:B at 25:75 (v/v). The column was maintained at ambient temperature. The flow rate was maintained at 250 μL/min, and a 20-μL injection was used. An API5000 triple quadrupole mass spectrometer (ABsciex, MA, USA) was used for detection, with electrospray ionization in positive ion mode. The mass spectrum parameters for the analytes were set as follows: ion spray voltage, 5500 V; curtain gas, 25 psi; ion source gas 1, 40 psi; ion source gas 2, 40 psi; source temperature, 500 °C; and collision gas, 5 psi. Multiple reaction monitoring transitions for baricitinib was m/z 307.3 → 186.0 and for ruxolitinib (internal standard) m/z 372.2 → 186.0. Analyst software version 1.5.2 was used to operate the mass spectrometer and to perform data analysis. Calibration curves were generated from standard of baricitinib by serial dilutions in blank mouse brain samples using the same extraction method described above. The calibration curves had *r*^2^ value greater than 0.99. The limit of detection of baricitinib was 5.5 ng/mL.

### Object recognition testing

On day 4 post-IC injection, the mice experienced 10 min of acclimatization to a Plexiglas chamber (60 cm × 60 cm). On day 5 post-injection, a “training phase” followed by a “preference test” after a delay of 5 min was performed on each mouse. In the training phase, duplicate copies of an object were placed near the two corners at opposite sides of the arena (15 cm from each adjacent wall). The animal was placed into the arena and allowed a total of 5 min of exploration of the two identical objects followed by removal from the arena. During the preference test or novel object testing (5 min duration on days 5 and 12), the mouse was placed back into the arena and presented with two objects: a familiar object from the training phase and a novel object. This process was repeated on day 6 post-infection, but with a 2-h delay (instead of 5 min on days 6 and 13) before the mouse was placed back into the arena. Treatments with baricitinib started after ORT on day 6 and continued until the mice were sacrificed on day 14. On day 12, mice underwent ORT identical to day 5 and on day 13 ORT was identical to day 6 (Fig. 1). Exploration times were recorded and used to calculate a discrimination index (DI, (time spent with object A minus time spent with object B)/(total time exploring both objects)) for the training and for the test sessions, respectively [9]. DI of 0 indicates equal exploration of both objects [15].

### In vitro treatment of monocytes or macrophages with baricitinib and HIV-1 for antiviral assays and activation markers

Mϕ and monocytes were isolated as previously described [8]. Cells were treated with various concentrations of baricitinib for 2 h prior to infection (HIV-1_baL_)_._ Cells were maintained for 6 days before viral quantification (p24-ELISA). For in vitro activation marker studies, CD14^+^ monocytes or fully differentiated (CD14^−^/CD11b^+^) Mϕ were used. Monocytes were used as a model to mimic HIV-induced activation that transpires in the periphery, wherein presence or internalization of HIV-1 in monocytes confers activation (CD14^+^/CD16^+^) and subsequent trafficking of “Trojan horse” monocytes across and within the BBB [11, 12]. The method used to differentiate Mϕ (CD14^−^/CD11b^+^) has been validated by our group to ensure that these cells are no longer expressing monocyte marker CD14 and are mature Mϕ (CD11b expression) [13, 17–20]. Mϕ or monocytes were treated with various concentrations of drug prior to infection (HIV-1_baL_) and maintained for 3 days before quantification of HLA-DR, CD163 (Mϕ), or CD14/CD16 (monocytes; Miltenyi Biotec, San Diego, CA). Mϕ and monocytes were treated with various concentrations of drug for 6 days and stained with Near-IR live/dead dye and quantified by FACS. Antiviral potency was calculated as previously described [21].

### Tissue preparation and staining

Fourteen days after IC MDM inoculation, mice were sacrificed and brain samples were harvested and placed in a tissue preservative solution (Miltenyi, Bergisch Gladbach, Germany). Samples were then dissociated using a commercial kit and a gentleMacs dissociator (Miltenyi) into single cell suspensions. Cells were then washed in 2% BSA/PBS buffer and incubated with the following: (1) anti-murine CD45-Vioblue (1:10 dilution) and anti-murine MHCII primary antibody (1:500) followed by APC conjugated secondary antibody (1:500 dilution), or isotype-only controls; (2) GFAP-FITC (1:750 dilution 1′ antibody and 1:1000 2′ 488 (FITC) antibody (Abcam, Cambridge, UK); or (3) human (non-murine cross-reactive) CD163-APC (Miltenyi Biotec) for 30 min (Sigma Aldrich, St. Louis, MO). Cells were washed twice in 2% BSA/PBS and fixed with 2% paraformaldehyde for 15 min. Cells were washed twice in 2% BSA/PBS and analyzed for double positive cells by flow cytometry (Macs Quant analyzer and MacsQuantify software, Miltenyi Biotec).

### Flow cytometry

Total events were collected using forward scatter (FSC) and side scatter (FSC) followed by doublet discrimination (FSC-area versus FSC-height) using a MacsQuant flow cytometer (Miltenyi). No primary FSC/SSC gate was established, to allow all events to be collected within the heterogeneous size and granularity of the brain cells. GFAP^+^ events were collected with gates and voltages established using 2′ antibody only. GFAP^+^ low granularity gate was established with events up to one-log shift from negative population, and GFAP^+^ high granularity (activated astrocytes) was established with events greater than two-log shift from negative population. MHCII and CD45 (mouse mononuclear phagocytes) expression were quantified based on quadrant gating for MHCII^+^/CD45^−^, CD45^+^MHCII^−^, MHCII^+^/CD45^+^, or MHCII^−^/CD45^−^. CD163^+^ events (human Mϕ) were collected with gates established from isotype-only controls. Percentages and total events were collected.

### Correlation matrices between behavior and brain markers of inflammation

Bivariate scatter plots of correlation data were created with Excel to visually and statistically assess correlations between inflammatory markers and object discrimination index on 13 (DI_13). Similar plots were also created to show correlations between different inflammatory markers. Fitted trendlines are displayed to show the degree and pattern of relationships. Coefficient of determination *r*^2^ and *p* values were calculated and displayed on the plots.

### Reservoir lifespan and expansion in macrophages

Primary human Mϕ were prepared as described above and infected with an MOI of 0.5 with HIV-1_BaL_ for 4 h prior to removal of virus and culture in virus-free medium. Supernatants were collected every 5 days, and extracellular p24 production was monitored (p24 ELISA, ABL, Inc.). Fresh culture medium was added to cultures every 5 days as supernatants were collected for p24 analysis. When extracellular p24 levels were below limit of detection (about 30 days post-infection), cells were incubated with various concentrations of baricitinib (0.001, 0.01, 0.1, 1.0, 10 μM), 3TC (negative control; same concentrations), or drug-free medium, for 2 h prior to addition of 50 ng/mL phorbol myristate acetate (PMA) to cultures to induce reactivation of latent HIV-1. After 24 h post-reactivation, cellular supernatants were collected, and extracellular reactivated p24 was quantified (p24 ELISA). Percent inhibition of reactivation was calculated versus no drug controls, and EC_50/90_ were calculated as previously described [21].

### In vitro treatment of monocytes or macrophages with baricitinib and HIV-1 for antiviral assays and activation markers

Primary human monocytes or Mϕ were isolated as described above and cultured in the following conditions: (1) drug-free medium and (2) 0.001, 0.01, 0.1, 1.0, or 10 μM baricitinib or 3TC (same concentrations) for 2 h prior to addition of HIV-1 BaL for 72 h. Cells were harvested and stained for CD14^+^ (FITC)/CD16^+^ (APC) to quantify HIV-induced activation or impact of drugs on block of these markers. Percent inhibition of reactivation was calculated versus no drug controls, and EC_50/90_ were calculated as previously described [21].

### In vitro cytotoxicity

The median inhibitory concentration (IC_50_) was determined by MTT (3-(4,5-dimethylthiazol-2-yl)-2,5-diphenyltetrazolium bromide) assay as previously described [21]. For all assays, cells were cultured as described above and maintained in various concentrations of drug-containing medium for 6 days prior to the assessment of toxicity. Cytotoxicity was considered when the concentrations of the test compounds alone inhibited viability by 50%. Additional negative control of cells incubated at various concentrations of 3′-azido-3′-deoxythymidine (AZT), which is known to be non-toxic to Mϕ and monocytes, was used as a negative control, and a dose response of cycloheximide (a protein synthesis inhibitor) was used as a cytotoxic control.

IC_50_ is a standardized pharmacological measure of in vitro cytotoxicity, as described mathematically and biologically, hence our choice for this method in this evaluation. Further, to underscore lack of toxicity at clinical concentrations and for concentrations used in this study, baricitinib is not toxic at concentrations more than 2 logs above what we report for concentrations in our murine model (Olumiant.com), underscoring that data observed are due to specific effects of baricitinib, not non-specific toxicity.

## Results

### Blood-brain barrier penetration

**Fig. 2:**
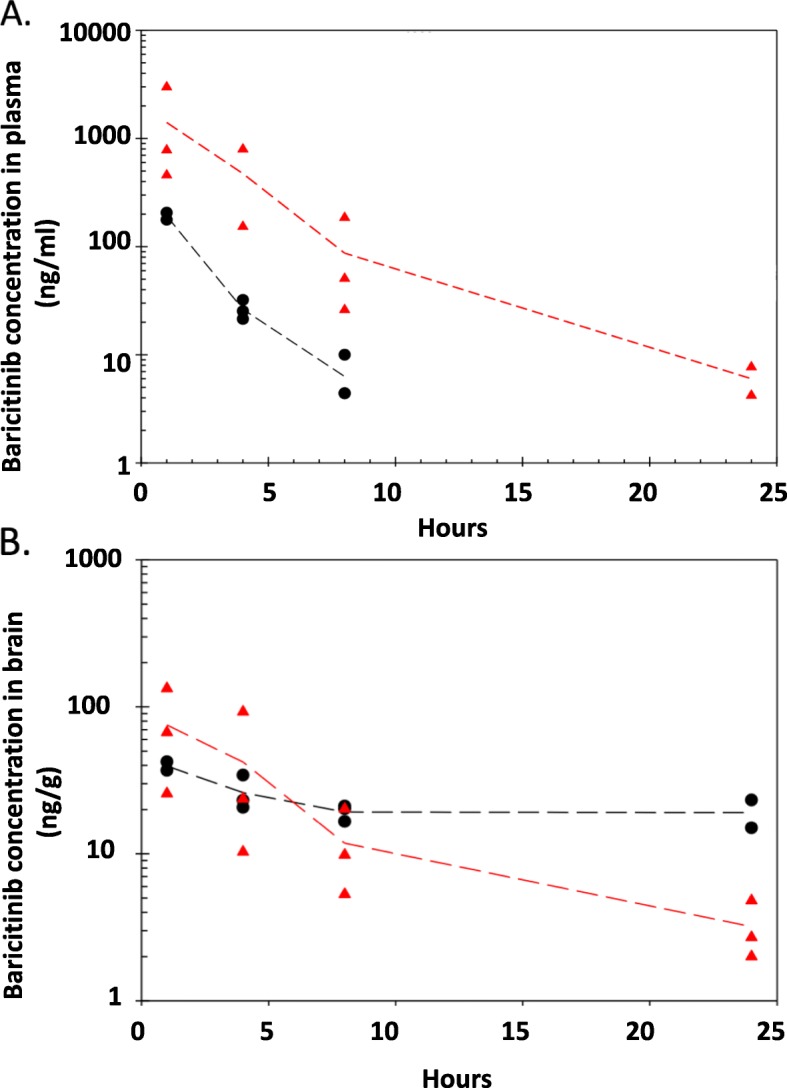
Pharmacokinetics of baricitinib in mice plasma or brains. Baricitinib concentrations in plasma (**a**, ng/mL) and brain (**b**, ng/g tissue), versus time after single SC of 10 (black circles) or 50 (red triangles) mg/kg. Each data point represents a mouse. Dashed curves were plotted through the averaged data from each time point

To better understand if baricitinib crosses the BBB, and to define dosing and pharmacokinetics of baricitinib in the brain, we sought to quantify the levels of baricitinib in mouse brains and plasma. Baricitinib was detected in all plasma and brain samples except for 24 h plasma samples at 10 mg/kg (Fig. 2a, b). Baricitinib C_max_ in plasma was about 200 and 1400 ng/mL for the 10 and 50 mg/kg doses, respectively, in approximately dose-proportional concentrations. Baricitinib can penetrate mouse BBB; Baricitinib C_max_ in the brain was about 40 and 75 ng/g for 10 and 50 mg/kg doses, respectively. Concentrations in the brain increased sublinearly with dose (Fig. 2b).

### Effect of baricitinib on object recognition testing

**Fig. 3:**
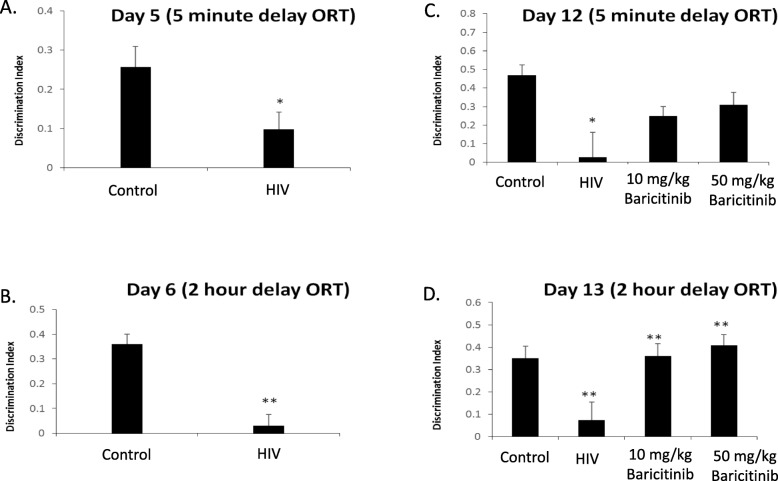
Baricitinib reverses behavioral abnormalities conferred by HIV in vivo. Discrimination index for novel object testing on days 5 and 6 (5 min delay or 2 h delay, **a** and **b** respectively) demonstrates that HAND (HIV) mice (untreated) cannot discriminate novel versus familiar object. However, uninfected control mice can discriminate. Day 12 and 13 ORT data demonstrate that uninfected mice can discriminate between novel and familiar objects; HIV infection significantly reduces ability of mice to discriminate between novel and familiar objects (**c**, **d**). Ten milligrams per kilogram and 50 mg/kg baricitinib restore the ability to discriminate to the level of the control mice (**c**, **d**). *p < 0.01, **p < 0.001. HAND (HIV) mice were compared to sham controls, and treated groups were compared to HAND (HIV) group

We sought to confirm that HAND mice demonstrate cognitive impairment (decreased object discrimination index) at both a 5-min and 2-h delay (Fig. 3a, b) prior to the addition of baricitinib. Further, we sought to determine if baricitinib can reverse existing ORT deficits in HAND mice, thereby representing a model where baricitinib could be used to treat existing HAND in humans.

By day 5 post-IC injection, control mice can discriminate novel versus familiar object after a 5-min delay, but HAND mice (untreated at this point) cannot (Fig. 3a). On day 6, control mice discriminate on a more difficult ORT (2 h delay) task, but untreated HAND mice did not discriminate (Fig. 3b). ORT abnormalities seen in HAND mice were reversed both at 10 mg/kg and 50 mg/kg baricitinib doses (quantified on days 12 and 13, after 6 and 7 days of baricitinib treatment; Fig. 3c, d).

### Effect of baricitinib on HAND markers in vivo

**Fig. 4:**
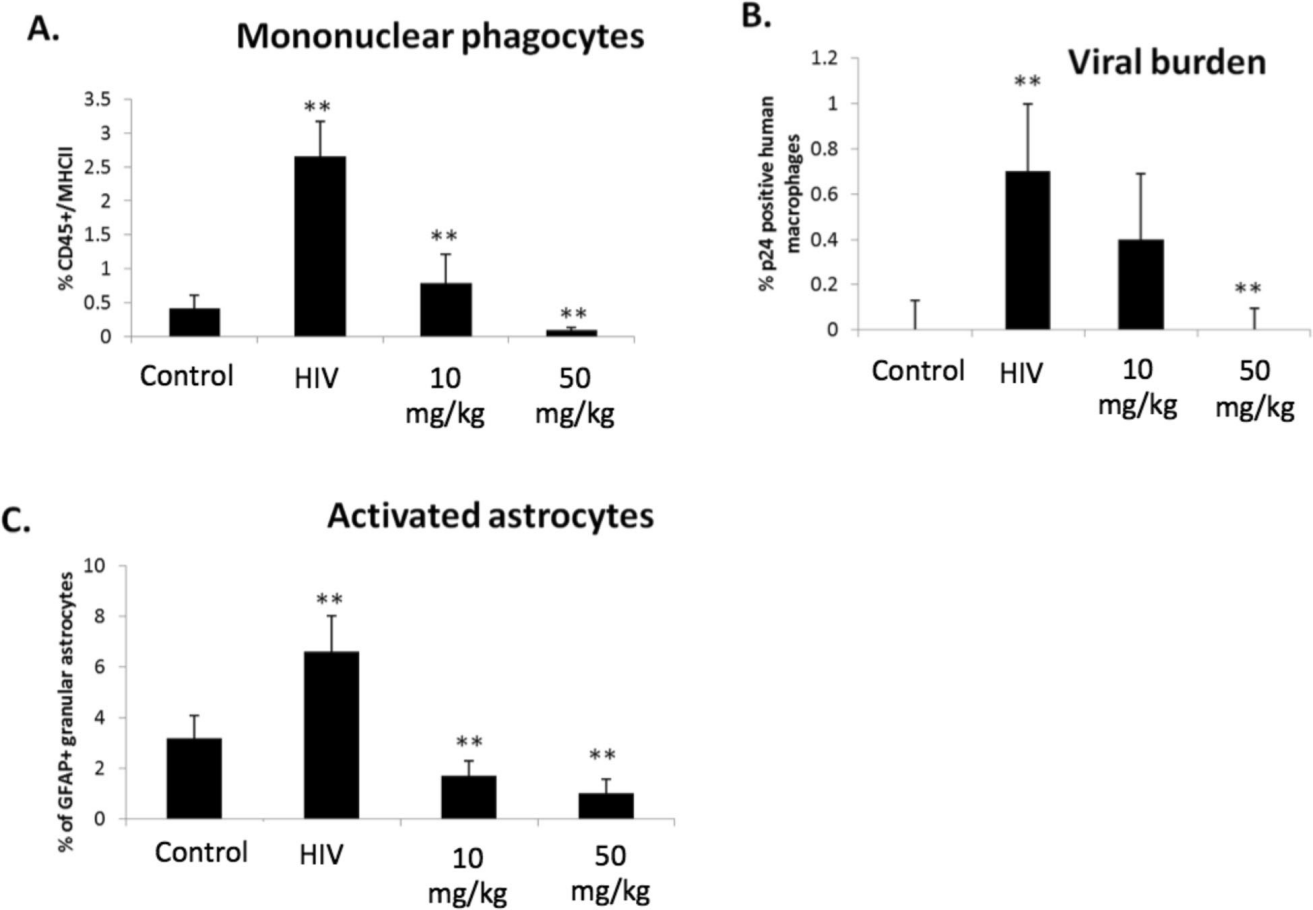
HIV induction of activation markers in vivo. Experimental mice were separated into four groups: control, HIV-infected, HIV-infected treated with low-dose baricitinib (10 mg/kg), and HIV-infected treated with high-dose baricitinib (50 mg/kg). Mice were sacrificed 14 days after infection, and brain samples were homogenized and assessed for encephalitis markers and viral protein using flow cytometry. **a** CD45/MHCII double staining as mononuclear phagocyte activation markers. **b** Activated p24+ human macrophages. **c** Activated astrocytes. **p < 0.001

We wanted to know if baricitinib could reverse phenotypic biomarkers of HAND including activated mononuclear phagocytes, astrogliosis, and activated human Mϕ containing HIV-1 (p24). Together, this information provides a foundation for understanding if a JAK inhibitor can reverse HIV-induced inflammation and activation that drives HAND in vivo. At 10 and 50 mg/kg, baricitinib significantly reduced the percentage of activated mouse mononuclear phagocytes (MHCII/CD45 double positive cells, Fig. 4a), high dose baricitinib significantly reduced activated p24^+^ human Mϕ (Fig. 4b; p24+ cells were also positive for CD163), and both baricitinib doses reduced activated astrocytes (GFAP^+^; astrogliosis, Fig. 4c) in the brain compared to untreated HAND mice. Data were corrected relative to isotype control, which was background subtracted from all samples including uninfected controls.

### Correlation analysis between baricitinib treatment, object recognition testing, and biomarkers of HAND

**Fig. 5:**
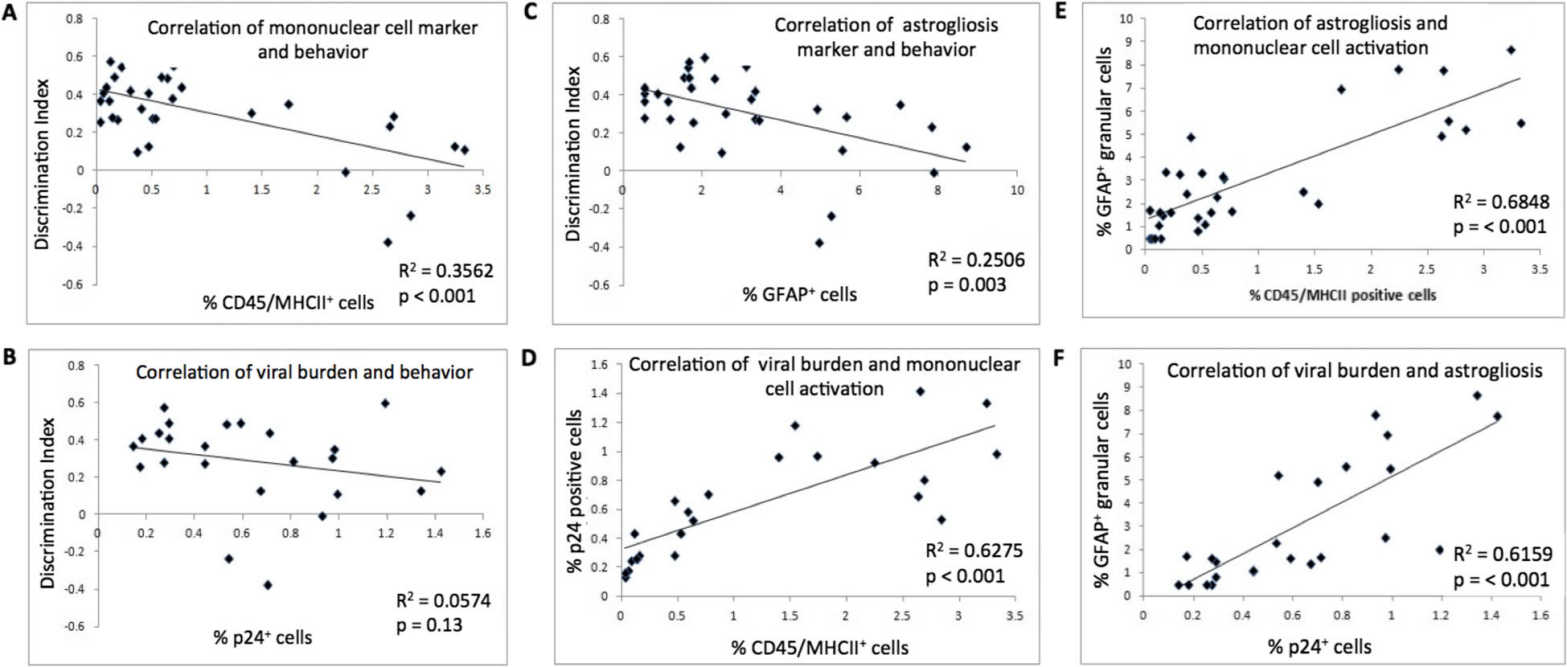
Correlation analysis of inflammatory markers with discrimination index and between different inflammatory markers. **a**–**c** Linear regression plots show correlations of discrimination index with different inflammatory markers. **a** Correlation of discrimination index with mononuclear cell activation markers with viral burden (**b**) and with astrogliosis marker (**c**). **d**–**f** Correlations between different inflammatory markers as well as viral burden. **d** Viral burden and mononuclear cell activation. **e** Astrogliosis and mononuclear cell activation. **f** Viral burden and astrogliosis

To better understand the link between activation markers and cognitive dysfunction conferred by HIV infection, we performed a correlation analysis between inflammatory markers and ORT, as well as between the different markers. A simple regression analysis showed a statistically significant correlation between markers of mouse brain activated mononuclear phagocytes and astrogliosis with cognitive dysfunction (*p* < 0.001 and *p* = 0.003, respectively; Fig. 5a, c). Cognitive dysfunction was not associated with viral burden (Fig. 5b).

Correlation analysis between the different inflammation markers and viral burden showed that activation of mouse mononuclear phagocytes and astrogliosis levels are highly correlated to the viral burden *p* < 0.001 (Fig. 5d, f). Mouse mononuclear cell activation was also strongly correlated with astrogliosis *p* < 0.001 (Fig. 5e).

### Cell-based antiviral potency

**Fig. 6:**
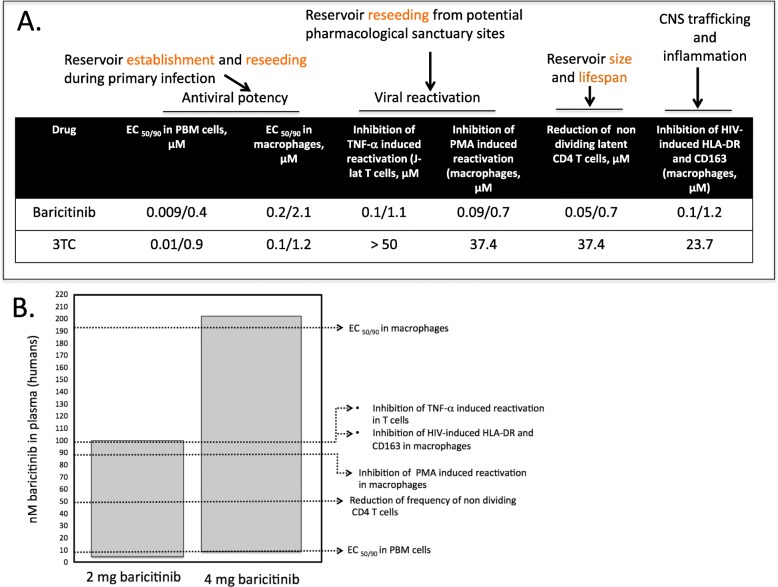
Baricitinib blocks reservoir establishment, maintenance, and expansion in primary human T cells and macrophages. Baricitinib demonstrates nanomolar inhibition of HIV replication in primary human macrophages and T cells, block of HIV-induced activation in macrophages, and HIV reactivation in T cells and macrophages (**a**). The concentrations that confer these anti-HIV effects are within the steady-state concentration range for approved doses of baricitinib in humans (**b**). Individual dose responses for data reported appear in Additional file 1: Figure S1

We sought to understand if JAK 1/2 inhibition by baricitinib could confer antiviral activity in clinically relevant cells involved in HIV persistence and activation that can promote CNS infection. Therefore, we measured the antiviral activity of baricitinib as previously described [22] in primary human Mϕ and activated peripheral blood mononuclear (PBM) cells. Baricitinib demonstrated submicromolar inhibition of HIV replication in primary human Mϕ and primary PBM cells (EC_50/90_ = 0.2 ± 0.1/2.1 ± 0.3 μM and 0.009 ± 0.003/0.4 ± 0.07 μM, respectively) (Fig. 6a).

### In vitro reservoir reseeding assays

We previously demonstrated that ruxolitinib and tofacitinib block the reactivation of latent HIV from T cells [23]. This block prevents reservoir reseeding that can promote spread of the viral reservoir both within T cells and to myeloid cells and can promote activation of cells that facilitate trafficking to the CNS [8, 11, 23–25]. Therefore, we measured the ability of baricitinib to block reactivation of latent HIV in T cells using the J-lat system. Baricitinib blocked the TNF-α-induced reactivation of HIV-1 in a T cell line of HIV reactivation (J-lat, with an EC_50/90_ 0.1 ± 0.08/1.1 ± 0.3 μM; Fig. 6a). Baricitinib also blocked the PMA-induced reactivation of HIV-1 from latent primary human Mϕ s, with an EC_50/90_ 0.09 ± 0.04/0.7 ± 0.1 μM, and reduced the frequency of non-dividing p24^+^ primary CD4^+^ T cells in vitro (EC_50/90_ 0.05 ± 0.02/0.7 ± 0.08 μM; Fig. 6a). The concentrations that conferred these anti-HIV effects are within the steady-state concentration range for approved doses of baricitinib in humans (Fig. 6b) [26, 27]. Individual dose responses for data appear in Additional file 1: Figure S1.

### In vitro activation marker assays

Chronic immune activation and inflammation persists even in individuals with well-controlled viremia, is a biomarker of HIV persistence, and correlates with non-AIDS-related morbidity and mortality in vivo [28, 29]. Additionally, cellular activation of T cells, monocytes, and Mϕ is correlated with HAND in vivo, and HAND is driven by an HIV-induced inflammatory state that persists even in the presence of cART. To date, no existing modalities are FDA approved to treat or reverse ongoing inflammation in HIV-infected individuals. We sought to measure the effect of baricitinib on HIV-induced activation of primary human Mϕ, a key cell type infected by HIV in the CNS. Baricitinib blocked the HIV-induced upregulation of activation markers HLA-DR and CD163 in primary human Mϕ (EC_50/90_ 0.1/1.2 μM; Fig. 6a, b; dose-response curves in Additional file 1: Figure S1).

## Discussion

A major finding of our work is that clinically relevant doses of baricitinib reverse behavioral abnormalities conferred by HIV and significantly reduce key pathological cell markers of HIVE, HIV persistence, and reservoir seeding in the CNS. These data demonstrate that baricitinib, when given systemically to HAND mice, crosses the BBB and has substantial and meaningful effects on important HAND pathological indices. Furthermore, the behavioral task is similar to episodic visual memory deficits noted in human HAND patients [10]. Additionally, concentrations of drug in the brain are sufficient to confer anti-inflammatory activity despite being lower than the plasma concentrations, and that the main message from these data is that baricitinib crosses the BBB and results in linear pharmacokinetic dynamics that suggests steady-state accumulation of drug. Linear pharmacokinetics in the brain, coupled with the observed phenotypic effects, demonstrates that baricitinib accumulates to sustained and adequate concentrations to confer anti-HIV and anti-inflammatory effects in the CNS compartment.

Since the deficits are relatively mild in HAND mice, they are comparable to milder forms of HAND in humans, and therefore, these improvements suggest baricitinib treatment in human HAND could reverse milder forms. Milder HAND is precisely the population that would have to be identified for a clinical trial and would potentially benefit most by adding an adjunctive treatment such as baricitinib to cART.

ORT abnormalities seen in HAND mice are reversed both at the 10 mg/kg and 50 mg/kg baricitinib doses and were coupled with a significant brain reduction in murine mononuclear phagocytes (CD45/MHCII positive cells). Activation of brain mononuclear phagocytes is probably the most important pathological marker of HAND in humans [18, 20, 24, 30, 31].

Human CD163 is a Mϕ activation marker, and activated Mϕ are correlated with increased inflammation in the CNS [8, 11, 12]. Baricitinib (at both doses) significantly decreased the frequency of p24^+^ human Mϕ compared to untreated HAND mice. Astrogliosis (GFAP^+^ cells) is conferred by HIV in the CNS, and increase in astrogliosis correlates with CNS inflammation in vivo [32–36]. Both baricitinib doses significantly decreased astrogliosis compared to untreated HAND mice. However, the data presented herein suggest that while virus may drive the glial (mononuclear phagocytes and astrocytes) reaction in HIVE, the amount of HIV in brain (i.e., p24+ cells) does not correlate with cognitive impairment in HAND mice (Fig. 5). One interpretation of this data, which is consistent with human pathological data from HAND patients, is that glial cells, particularly microglia [30, 31], are driving cognitive dysfunction in HAND mice and in humans. Since JAK inhibitors like baricitinib inhibit mononuclear phagocyte functions and also reverse cognitive dysfunction in HAND mice, these data further link mononuclear phagocyte activation to the pathogenesis of HAND and further suggest that the HAND mouse model used in these studies is highly relevant.

It is beyond the scope of this article to provide a comprehensive comparison of HAND models. Though our SCID mouse model has arguable limitations, it shows a strong correlation between activated mouse mononuclear phagocytes in the brain and behavioral deficits measured by ORT. This reflects a clinically relevant correlation between mild HAND symptoms and activated mononuclear phagocytes in the brain of humans with HAND [20, 24, 30, 31]. However, our reported model uses uninfected (controls) and HIV-infected human MDM, which are IC injected into mice, in order to simulate HIV-induced brain pathology. These xenografts are relatively short-lived (approximately 6 weeks), and therefore, this model does not allow one to study CNS invasion of HIV and long-term effects of brain HIV infection. Other models of HAND, such as the SIV encephalitis, the neuro-invasive BLT model, and the EchoHIV model, reflect important aspects of human HAND, but also have limitations [37–40].

In vitro experiments in primary human Mϕ demonstrated that baricitinib confers submicromolar inhibition of HIV-1 replication, HIV-induced activation, and block of reservoir establishment, maintenance, and expansion. These effects are conferred at concentrations found within the steady state in humans [26, 27] and baricitinib package insert (Olumiant.com). In addition, the data demonstrates the ability of baricitinib to reduce the number of activated murine mononuclear phagocytes (CD45/MHCII double positive cells), activated human MΦ, p24^+^ cells, and HIV-induced astrogliosis in the brains of HAND mice, which reflects JAK inhibition of those cells. Therefore, JAK activation in mononuclear phagocytes is associated with neurotoxicity in the presence of HIV infection. Consequently, JAK inhibitors could be used adjunctively with cART in subjects who are diagnosed to have HAND, addressing an unmet, important clinical need that is not addressed by cART alone. Data from the AIDS Clinical Trial Group using a JAK inhibitor, ruxolitinib, in HIV-infected patients on cART is currently being analyzed and should inform about safety and other potential positive effects (ClinicalTrials.gov). Our group recently reported that the ruxolitinib is safe and well-tolerated in HIV-infected, cART-suppressed individuals, with no adverse effects versus control cART group. Further, a significant reduction in sCD14, a clinical marker for monocyte activation, was observed in the ruxolitinib arm versus cART group [14]. This study is not addressing the effects of ruxolitinib on HAND. In anticipation of favorable results of ruxolitinib, our studies provide convincing impetus to consider a phase II trial of baricitinib, which is better tolerated than ruxolitinib, and provides once a day dosing, in HAND individuals on cART. We anticipate that baricitinib will improve cognitive functions, as well as HIV load and systemic inflammatory markers, in these individuals and thus represent an important step in treating a serious problem that affects 50% of HIV-infected individuals.

## Conclusion

Our group recently reported the safety and tolerability of ruxolitinib in HIV-infected individuals [14], underscoring the potential safety and utility of JAK inhibitors for additional human trials. Our in vivo studies have shown that baricitinib, an FDA-approved JAK inhibitor, crosses the BBB and ameliorates cognitive dysfunction in HAND mice. Baricitinib treatment of HAND mice also reduces brain HIV and curtails the neuroinflammatory markers of HAND. In vitro studies showed that baricitinib significantly reduced the markers of persistence, reservoir size, and reseeding in Mϕ. Given that HAND afflicts approximately 50% of cART-suppressed HIV patients, it is important to develop adjunctive, novel treatments. The data reported herein coupled with our recent human trial with JAK inhibitors provide compelling preclinical data and impetus for considering a trial of baricitinib in HAND individuals treated with cART to reverse cognitive deficits and key events driving viral persistence.

## Additional file

Additional file 1: **Figure S1.** Dose response curves for anti-HIV effects of baricitinib in vitro. Graphs A-F represent dose response curves for EC50/90 data reported in Fig. 6. For all graphs, baricitinib data are plotted in dotted lines with triangles, and 3TC data are plotted in solid lines with circles. 3TC was evaluated as a control for each dose response. Antiviral effect of baricitinib in PBM cells and macrophages (A, B respectively). Inhibition of TNF-a induced reactivation in J-lat T cells appear in (C), and inhibition of PMA induced reactivation in macrophages appear in (D). Reduction of the frequency of non-dividing latent CD4 T cells appear in (E), and reduction of HIV-induced activation markers HLA-DR/CD163 double positive macrophages appear in (F). For all assays, baricitinib demonstrated a dose dependent reduction in pro-HIV events. As expected 3TC reduced viral replication in PBM cells and macrophages (A, B), but did not have any effect on inflammatory or latency events (C-F). The *n* = 3 independent experiments conducted with 4 pooled donors per experiment. (PPTX 93 kb)

## Data Availability

Not applicable.

## Abbreviations

BBB: Blood-brain barrier
cART: Combined antiretroviral therapy
CNS: Central nervous system
GFAP: Glial fibrillary acidic protein
HAND: HIV-associated neurocognitive disorder
HIVE: HIV encephalitis
MDM: Monocyte-derived macrophages
Mϕ: Macrophages
ORT: Object recognition testing
