# Supplementary material for: Baricitinib reverses HIV-associated neurocognitive disorders in a SCID mouse model and reservoir seeding in vitro
Source: J Neuroinflammation. 2019 Sep 27;16:182. doi: 10.1186/s12974-019-1565-6 (PMC6764124; doi:10.1186/s12974-019-1565-6)
Supplement: Supplementary file 1 — Figure S1. Dose response curves for anti-HIV effects of baricitinib in vitro. Graphs A-F represent dose response curves for EC50/90 data reported in Fig. 6. For all graphs, baricitinib data are plotted in dotted lines with triangles, and 3TC data are plotted in solid lines with circles. 3TC was evaluated as a control for each dose response. Antiviral effect of baricitinib in PBM cells and macrophages (A, B respectively). Inhibition of TNF-a induced reactivation in J-lat T cells appear in (C), and inhibition of PMA induced reactivation in macrophages appear in (D). Reduction of the frequency of non-dividing latent CD4 T cells appear in (E), and reduction of HIV-induced activation markers HLA-DR/CD163 double positive macrophages appear in (F). For all assays, baricitinib demonstrated a dose dependent reduction in pro-HIV events. As expected 3TC reduced viral replication in PBM cells and macrophages (A, B), but did not have any effect on inflammatory or latency events (C-F). The n = 3 independent experiments conducted with 4 pooled donors per experiment. (PPTX 93 kb) [file 12974_2019_1565_MOESM1_ESM.pptx]

## Slide 1
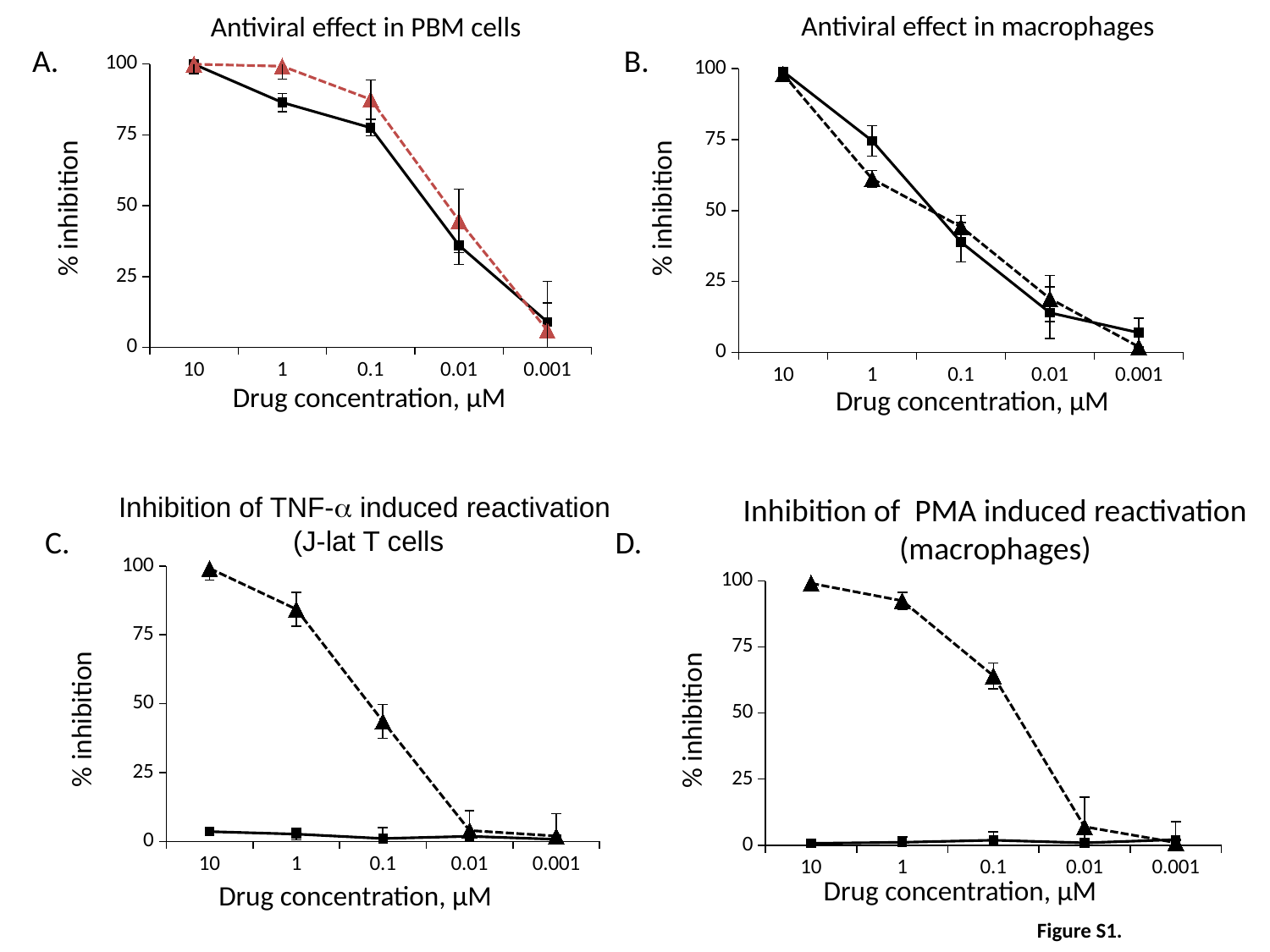

Antiviral effect in macrophages
Antiviral effect in PBM cells
A.
B.
### Chart
| Category | 3TC | Baricitinib |
|---|---|---|
| 10 | 99.9 | 99.9 |
| 1 | 86.4 | 99.2 |
| 0.1 | 77.5 | 87.5 |
| 1.0000000000000005E-2 | 36.0 | 44.7 |
| 1.0000000000000007E-3 | 9.0 | 6.0 |
### Chart
| Category | 3TC | Baricitinib |
|---|---|---|
| 10 | 99.0 | 98.1 |
| 1 | 74.6 | 61.2 |
| 0.1 | 38.9 | 44.3 |
| 1.0000000000000005E-2 | 14.0 | 19.0 |
| 1.0000000000000007E-3 | 7.0 | 2.0 |% inhibition
% inhibition
Drug concentration, µM
Drug concentration, µM
Inhibition of TNF-a induced reactivation
(J-lat T cells
Inhibition of PMA induced reactivation (macrophages)
C. D.
### Chart
| Category | 3TC | Baricitinib |
|---|---|---|
| 10 | 3.6 | 99.1 |
| 1 | 2.7 | 84.3 |
| 0.1 | 1.1 | 43.6 |
| 1.0000000000000005E-2 | 1.9000000000000001 | 4.0 |
| 1.0000000000000007E-3 | 0.8 | 2.0 |
### Chart
| Category | 3TC | Baricitinib |
|---|---|---|
| 10 | 0.7000000000000003 | 99.1 |
| 1 | 1.1 | 92.5 |
| 0.1 | 1.9000000000000001 | 64.1 |
| 1.0000000000000005E-2 | 0.9 | 7.0 |
| 1.0000000000000007E-3 | 2.1 | 1.0 |% inhibition
% inhibition
Drug concentration, µM
Drug concentration, µM
Figure S1.

## Slide 2
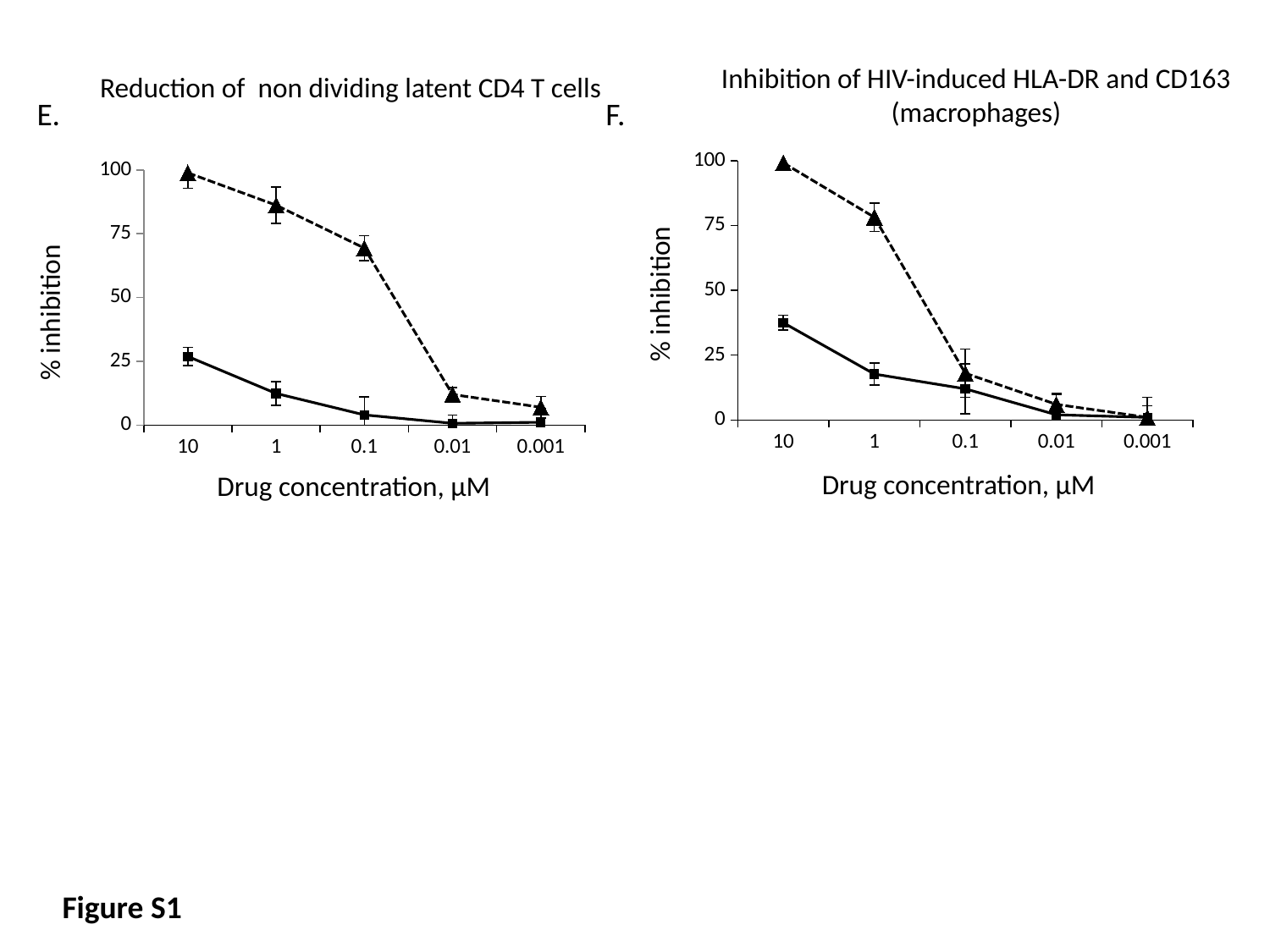

Inhibition of HIV-induced HLA-DR and CD163 (macrophages)
Reduction of non dividing latent CD4 T cells
E. F.
### Chart
| Category | 3TC | Baricitinib |
|---|---|---|
| 10 | 37.5 | 99.2 |
| 1 | 17.7 | 78.2 |
| 0.1 | 12.0 | 18.0 |
| 1.0000000000000005E-2 | 2.0 | 6.0 |
| 1.0000000000000007E-3 | 1.0 | 1.0 |
### Chart
| Category | 3TC | Baricitinib |
|---|---|---|
| 10 | 26.9 | 98.9 |
| 1 | 12.4 | 86.2 |
| 0.1 | 4.0 | 69.4 |
| 1.0000000000000005E-2 | 0.7000000000000003 | 12.0 |
| 1.0000000000000007E-3 | 1.1 | 7.0 |% inhibition
% inhibition
Drug concentration, µM
Drug concentration, µM
Figure S1
